# Supplementary material for: The interactome of intact mitochondria by cross-linking mass spectrometry provides evidence for coexisting respiratory supercomplexes
Source: Mol Cell Proteomics. 2017 Dec 8;17(2):216–32. doi: 10.1074/mcp.RA117.000470 (PMC5795388; doi:10.1074/mcp.RA117.000470)
Supplement: Supplemental Data [file supp_RA117.000470_133922_0_supp_23978_fzffwf.zip › spectra_annotation/mito_spectra_annotation/285-1-2-1-12-1.pdf]

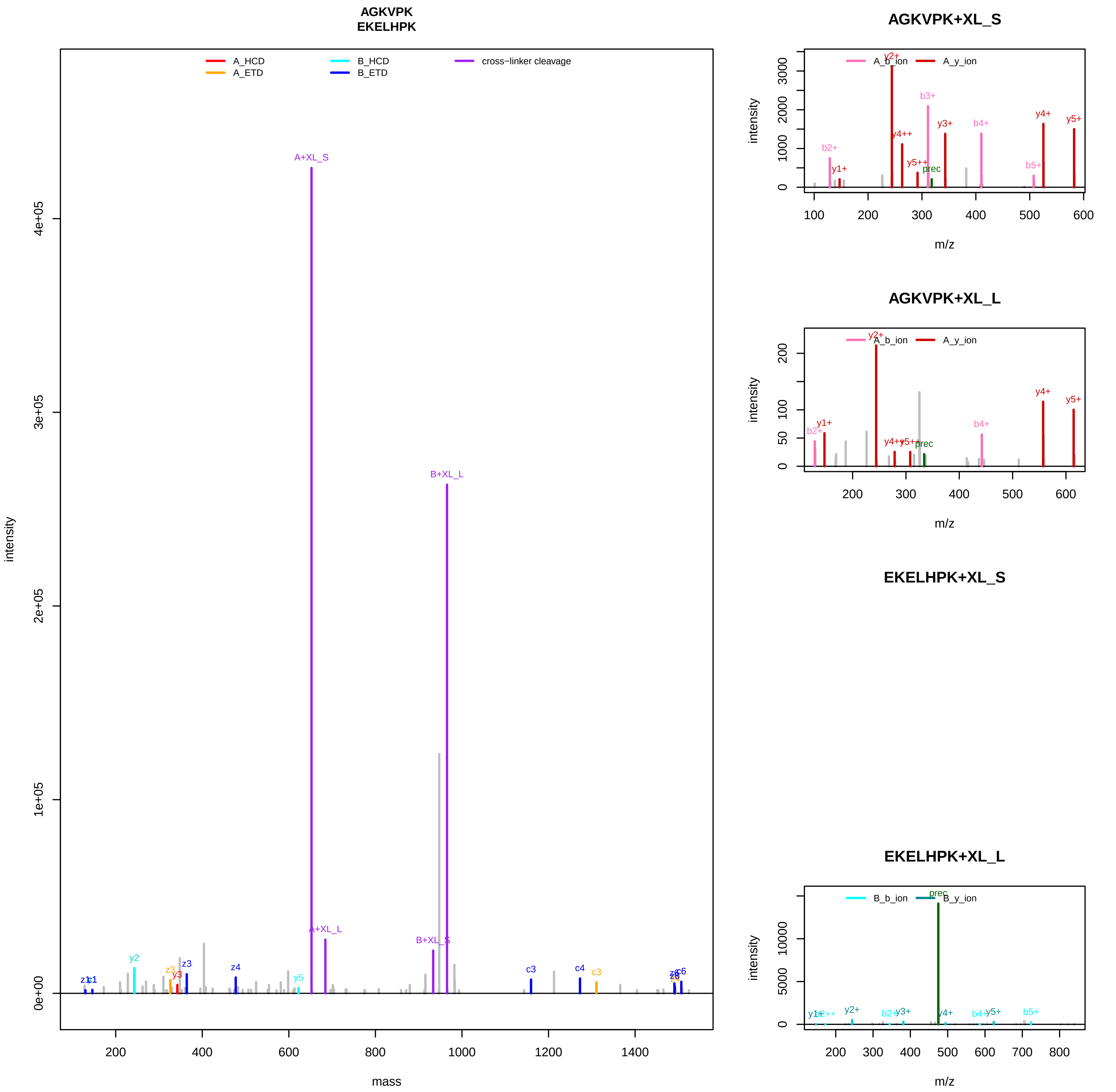

AGKVPK
EKELHPK
A_HCD
A_ETD
B_HCD
B_ETD
cross−linker cleavage
A+XL_S
B+XL_L
A+XL_L
B+XL_S
intensity
mass
0e+00
1e+05
2e+05
3e+05
4e+05
200
400
600
800
1000
1200
1400
y2
z3
y3
z4
y5
c3
c4
c6
z1
c1
AGKVPK+XL_S
A_b_ion
A_y_ion
y2+
b3+
y3+
b4+
y4+
y5+
b2+
y1+
y4++
y5++
prec
b5+
m/z
0
1000
2000
3000
100
300
500
600
AGKVPK+XL_L
50
100
200
EKELHPK+XL_S
EKELHPK+XL_L
B_b_ion
B_y_ion
5000
10000
700
